# Effect of pharmacogenetic variations on praziquantel plasma concentration and safety outcomes among school children in Rwanda

**DOI:** 10.1038/s41598-023-28641-w

**Published:** 2023-01-26

**Authors:** Abbie Barry, Joseph Kabatende, Nigus Fikrie Telele, Rajabu Hussein Mnkugwe, Michael Mugisha, Lazare Ntirenganya, Emile Bienvenu, Eleni Aklillu

**Affiliations:** 1 Division of Clinical Pharmacology, Department of Laboratory Medicine, Karolinska Institutet at Karolinska University Hospital, Huddinge, Stockholm, Sweden; 2 Rwanda Food and Drugs Authority, Nyarutarama Plaza, KG 9 Avenue, Kigali, Rwanda; 3 Department of Clinical Pharmacology, School of Medicine, Muhimbili University of Health and Allied Sciences, Dar es Salaam, Tanzania; 4 College of Medicine and Health Sciences, University of Rwanda, KK 737, Kigali, Rwanda

**Keywords:** Infectious diseases, Genotype, Metabolism

## Abstract

School-based mass drug administration (MDA) of Praziquantel (PZQ) is the global intervention strategy for elimination of schistosomiasis. Genetic variations in drug metabolizing enzymes and transporter proteins influences drug exposure and treatment outcomes, but data on PZQ pharmacokinetics and safety outcomes are scarce. We investigated the effect of pharmacogenetics variations on PZQ plasma concentrations and safety outcomes among 462 Rwandan schoolchildren who received single dose PZQ and albendazole in MDA. Genotyping for common functional variant alleles CYP3A4*1B, *CYP3A5* (*3, *6, *7), *CYP2C19* (*2, *3, *17), *CYP2C9* (*2, *3) and CYP2J2*7 were done. Plasma concentration of PZQ, *cis*-4-OH-PZQ and *trans*-4-OH-PZQ were measured using LC/MS/MS. Active safety monitoring was done on days 1, 2, and 7 post-MDA. *CYP2C9* and *CYP2C19* genotypes were significantly associated with PZQ plasma concentrations and its *cis*- and *trans*-4-OH-PZQ/PZQ metabolic ratios (MR). CYP2C9*2 and CYP2C9*3 carriers had significantly higher PZQ concentration (p = 0.02), lower *trans*-4-OH-PZQ/PZQ (p < 0.001), and *cis*-4-OH-PZQ/PZQ (p = 0.02) MR. *CYP2C19* (*2, *3) carriers had significantly higher plasma PZQ concentration than CYP2C19 *1/*1 and CYP2C19 *17 carriers (*1/*17 or *17/*17) (p < 0.001). *CYP3A4* was significantly associated with cis-4-OH-PZQ MR (p = 0.04). Lower *cis*-4-OH-PZQ/PZQ MR (p < 0.0001) was a predictor of MDA-associated adverse events, but no significant association with genotypes were found. In conclusion, *CYP2C9* and *CYP2C19* genotypes significantly influence the plasma PZQ concentration and its MR. Lower cis-4-OH-PZQ/PZQ MR is significant predictor of adverse events following MDA.

## Introduction

Praziquantel (PZQ) is the only World Health Organization (WHO) approved drug for the treatment and prevention of *Schistosoma haematobium* (urogenital schistosomiasis) and *Schistosoma mansoni* (intestinal schistosomiasis) currently^1^. Schistosomiasis is among the most prevalent neglected tropical diseases and public health problem in many parts of the world particularly in sub-Saharan Africa (SSA)^2^. More than 90% of all schistosomiasis cases are from SSA^3^. Approximately 20 million people suffer from schistosomiasis related complications and the infection causes up to 280,000 deaths annually^4,5^. The WHO recommends preventive chemotherapy of PZQ as a global intervention strategy to reduce transmission, control and eliminate schistosomiasis as a public health problem in endemic countries^5^. According to the WHO, in 2019, it was estimated that 236.6 million people required preventive treatment for schistosomiasis and more than 105.4 million people were reportedly treated^2^.

In settings where both schistosomiasis and soil transmitted helminths are endemic, single dose 40 mg/kg body weight of PZQ is co-administered with albendazole during mass drug administration (MDA)^6^. School children are higher risk of schistosomiasis and STH infections, hence the target population for preventive chemotherapy^7,8^. Although, mass PZQ administration played a significant role in reducing disease-associated morbidity and mortality, the disease remains endemic in many countries in SSA including Rwanda^9–11^. In Rwanda, transmission of intestinal schistosomiasis is prevalent, and children aged 5–10 years are the most affected^8,12^. Although mass PZQ and albendazole co-administration is generally safe, approximately 20% of children in Rwanda experienced mild to severe adverse events post MDA^13^.

Studies on other infectious diseases including malaria, HIV and Tuberculosis found that pharmacogenetic variation influences plasma drug exposure and treatment outcomes including safety^14–18^. Pharmacogenetic variations in drug metabolizing enzymes and transporter proteins relevant for PZQ disposition may have an impact on plasma concentration and treatment outcomes such as safety. PZQ is primarily metabolized by CYP450 enzymes, including *CYP3A4*, *CYP3A5*, *CYP2C19* and *CYP2C9*^19^ to produce several metabolites, including 4-OH-PZQ (*trans*- and *cis*-), a major metabolite of PZQ in human^20^. Most of these enzymes relevant for PZQ disposition are genetically polymorphic, displaying interindividual variability in enzyme activity^21^. The change in enzyme activity due to pharmacogenetic variations may cause inter-individual variability in plasma drug exposure and safety outcome (treatment-associated adverse events)^21^. Previous pharmacokinetics and pharmacodynamic studies have indicated that high plasma drug exposure may increase the risk of adverse events^15,17,22^. Therefore, genetic variations in CYP enzymes relevant for PZQ biotransformation may affect both PZQ plasma exposure and safety^20^. To date, few studies have investigated the role of pharmacogenetics on the pharmacokinetics of PZQ. Studies investigating the influence of genetic variations on PZQ plasma concentration are lacking^23^. To the best of our knowledge, only two studies investigated the pharmacogenetics variations of PZQ and its relevance on plasma concentration and schistosomiasis treatment outcomes among Schistosomiasis infected children in Tanzania and Zimbabwe^20,24^. The authors reported relevance of *CYP2C19* genotype for PZQ concentration and *trans*-4-OH-PZQ/PZQ ratio and a borderline association between *CYP3A5* genotype and treatment-associated adverse events^20^. More studies are needed to characterize the importance of pharmacogenetic variations for PZQ plasma exposure and treatment outcome since the populations differ partly due to wide genetic diversity among black African populations^17,25^. Our study is the first to investigate the effect of pharmacogenetics on PZQ concentration and safety outcome among children who received PZQ as part of MDA. Additionally, the effect of *CYP2J2* genotype on PZQ concentration and the effect of pharmacogenetic variation on *cis*-4-OH-PZQ concentration and its metabolic ratio (MR), *cis*-4-OH-PZQ/PZQ have not been investigated.

The utility of pharmacogenetic data to improve treatment outcomes has recently been intensified in Africa^26^. However, the influence of pharmacogenetic variations on the pharmacokinetics and safety outcome of drugs used in MDA campaigns remains to be investigated. Even though it is a challenge to implement individualized treatment during MDA, understanding how genetic variations impact the pharmacokinetics and treatment associated adverse events of drugs used in MDA is essential for improving treatment outcomes in the future^14,20,27^. Therefore, the objective of this study was to investigate the effect of pharmacogenetics variations on PZQ plasma concentrations and safety outcomes among school children who received PZQ co-administered with albendazole in Rwanda.

## Materials and methods

### Study design, setting and population

This pharmacogenetics, pharmacokinetics, and pharmacodynamics (safety) prospective cohort study included 462 school children aged 5–15 years attending six schools in three districts located around the belt of Lake Kivu, namely Rubavu, Nyamasheke, and Rusizi in the Western Province of Rwanda. The three districts are endemic for intestinal schistosomiasis and soil transmitted helminths. This study received ethical approval from Rwandan National Ethics Committee and Karolinska Institutet, and was conducted in accordance with the principles outlined in the Declaration of Helsinki.

Data including sociodemographic, anthropometrics (height, weight) and pre-MDA symptoms for safety assessment were collected using a case record form administration through interviews. Anthropometric measurements, children’s body weight was measured in kilograms (kg) and height was measured in centimeters (cm), and then converted to body mass index (BMI)-for-age Z score (BAZ) and height-for-age Z score (HAZ), respectively, using WHO Anthro-Plus software for school-age children^28^. Children who had HAZ and BAZ scores < 2 standard deviations (SD) were considered stunted and wasted/thin, respectively.

### Preventive chemotherapy and adverse event monitoring

All participants were interviewed for any pre-existing clinical symptoms (pre-MDA event), such as fever, loss of appetite, dizziness or fainting, confusion, drowsiness, headache, cough, difficulty in breathing, nausea, vomiting, diarrhea, stomach pain, itching, rash, and any other symptoms. Study participants received single dose albendazole and PZQ in MDA campaigns as preventive chemotherapy provided through the Rwandan national NTD program. All children were informed to eat food before coming to school to participate in the MDA campaign. Number of PZQ tablets was based on height of children (≥ 94 cm dose pole, designed to deliver a dose of at least 40 mg/kg) and albendazole 400 mg following the national and WHO MDA guidelines^6,29^. The Rwanda NTD public health program of the Ministry of Health provided and co-administered PZQ and albendazole as preventive chemotherapy to prevent transmission and control of schistosomiasis and STH.

Following MDA, study participants were actively monitored to record MDA-related adverse events (AEs) on days 1, 2, and 7 post MDA^13^. Collected data were entered into an electronic database using tablets and cleaned before statistical analysis. To distinguish between pre-existing clinical symptoms and treatment-related adverse events (AEs) following PZQ and albendazole MDA, events reported by each study participant before and after MDA were cross-checked and verified^13^. Treatment-associated adverse events (AEs) were defined as any medical event (sign or symptom) that occurred after drug intake (post-MDA) and the same type of events were not reported prior to the administration of the drug (pre-MDA)^13,30,31^.

### Quantification of praziquantel, *cis*-4-hydroxy-praziquantel and *trans*-4-hydroxy-praziquantel plasma concentrations

Reference standards such as racemic PZQ, *trans*-4-OH-PZQ, and *cis*-4-OH-PZQ and their respective internal standards rac-PZQ-d11, *trans*-4-OH-PZQ-d5, and *cis*-4-OH-PZQ-d5 were purchased from Toronto Research Chemicals (Toronto, Ontario, Canada). Acetonitrile, methanol, and formic acid of mass spectrometry (MS) grade were purchased from Merck (Darmstadt, Germany). Ultra-pure MilliQ water was prepared using a Milli-Q water purification system (Merck Millipore, Massachusetts, USA). Blank plasma was supplied by the blood bank of the Karolinska University Hospital Huddinge (Stockholm, Sweden).

Whole blood sample was collected 2 h post MDA from all 462 study participants in heparinized tubes and immediately centrifuged at 1000 rpm for 10 min and plasma aliquot was kept in − 80 °C freezer until analysis. The plasma concertation of PZQ, *trans*-4-OH-PZQ and *cis*-4-OH-PZQ using Ultra high Performance Liquid Chromatography tandem–Mass Spectrometry (UPLC-MS/MS) as described previously^20^. In brief, 100 µL of plasma sample went through protein precipitation with 300 µL of internal standards solution containing 50 ng/mL each of rac-PZQ-d11, trans-4-OH-PZQ-d5, *cis*-4-OH-PZQ-d5 in a 50:50 mixture (v:v) of acetonitrile:methanol. The mixture was vortexed for 3 min and centrifuged for 20 min at 3220 g at 4 °C. Thereafter, 75 µL of the supernatant was diluted with 75 µL MilliQ water, and 5 µL of the mixture was injected into the UPLC-MS/MS system. Standards and quality control (QC) samples were prepared in the same manner by adding 10µL standard and QC 10× concentrated solutions to 90 µL blank plasma.

The calibration curves were constructed within the range of 2.4 to 2500 ng/mL for PZQ and *cis*-4-OH-PZQ. For trans-4-OH-PZQ, the range of the calibration curve was constructed within 24 to 25,000 ng/mL. About 7–9 calibration points were injected twice before the samples and once after. The QC samples were injected for every 20 samples. Quantification of the analytes was performed using the analyte to internal standard integrated peak area ratio with the MassLynx application manager TargetLynx (Waters). *Trans*-4-OH-PZQ d5 was used as internal standard also for cis-4-OH-PZQ since their retention times were similar. Quality control samples at 9.8, 78.1, and 1250 ng/mL were injected at regular intervals during each analysis.

### Genotyping for *CYP3A4*, *CYP3A5*, *CYP2C19*, *CYP2C9* and *CYP2J2*

Genomic DNA was extracted from whole blood using the QIAamp DNA Midi Kit (Qiagen GmbH, Germany) as per the manufacturer’s instruction. Genotyping for CYP3A4*1B, CYP3A5*3, CYP3A5*6, CYP3A5*7, CYP2C19*2, CYP2C19*3, CYP2C19*17, CYP2C9*2, CYP2C9*3, and CYP2J2*7 was done as previously described^14,20^. Briefly, genotyping was performed using TaqMan® drug metabolism genotyping assay reagents for allelic discrimination (Applied Biosystems Genotyping Assays) with the following ID numbers for each SNP: C__11711730_20 for CYP3A4*1B (− 392A>G, rs2740574), C__26201809_30 for CYP3A5*3 (c.6986A4G, rs776746), C__30203950_10 for CYP3A5*6 (g.14690G4A, rs10264272), C__32287188_10 for CYP3A5*7 (g.27131_27132insT rs41303343), C__25986767_70 for CYP2C19*2 (rs4244285), C__2,7861809_10 for CYP2C19*3 (rs4986893), C__469857_10 for CYP2C19*17 (rs12248560), C__25625805_10 for CYP2C9*2 (rs1799853), C__27104892_10 for CYP2C9*3 (rs1057910) and C__9581699_80 for CYP2J2*7 (rs890293). Genotyping was done using 7500 Fast Real-Time PCR (Applied Biosystems, United States). The final volume for each reaction was 10 μL, consisting of 1 μL genomic DNA and 9 μL TaqMan fast advanced master mix (Applied Biosystems, Waltham, MA, United States), DNA/RNA free water and TaqMan drug metabolism genotyping assays mix (Applied Biosystems). The PCR consisted of an initial step at 60 °C for 30 s, hold stage at 95 °C for 10 min and PCR stage for 40 cycles step 1 with 95 °C for 15 and step 2 with 60 °C for 1 min and after read stage with 60 °C for 30 s.

### Statistical analysis

Socio-demographic characteristics were summarized as proportions for categorical data and median (range or Interquartile range- IQR). The *CYP3A4* (*1/*1 or *1B carrier), *CYP3A5* (*1/*1 or *3, *6 *7 carriers), *CYP2C9* (*1/*1 or *2, *3 carriers) and *CYP2J2* (*1/*1 or *7 carriers) genotypes were dichotomized as variant allele carriers and non-carriers (wild type). *CYP2C19* genotype was categorized as Ultrarapid Metabolizer (*1/*17 and *17/*17), Extensive Metabolizers (*1/*1), and intermediate or poor metabolizers (*2, *3 carriers). Chi-square test was used to compare the genotype and allele frequencies between the observed and expected according to the Hardy–Weinberg equilibrium.

Plasma concentration of PZQ and its MRs were transformed to log10 values before statistical analysis. Independent t-test or one-way ANOVA was used to compare the geometric means of PZQ, *cis*-4-OH-PZQ/PZQ and trans-4-OH-PZQ/PZQ MR concentrations between genotypes. A univariate followed by multivariate linear regression analysis were used to identify the predictors of PZQ plasma concentrations. Independent samples t-test was used to compare the geometric means of PZQ and its trans and cis-4-OH MRs among those who experienced any adverse event and those who did not experience adverse events. Chi-square or fisher’s exact tests was used to test for associations between adverse events following MDA and genotypes. Univariate and multivariate log binomial regression was used to quantify the effect of association between the different genotypes and adverse event.

### Ethical approval

This study received ethical approval from the Rwandan National Ethics Committee (Review Approval Notice No. 0064/RNEC/2019) and Stockholm Ethics Committee, Sweden (Ref.No. 2020-00845). Prior to enrolment, participants and their parents or legal guardians received information about the study. For participants ≤ 12 years of age, verbal and written informed consent was obtained from their parent or guardian, and for participants > 12 years of age, verbal and written informed consent was obtained from the parent or guardian and assent was obtained from the study participant.

## Results

### Sociodemographic and nutritional status of study participants

A total of 462 school children who received PZQ and albendazole MDA were enrolled in this study, of whom 50.4% were females. The median age (interquartile) of the study cohort was 12 (10–13) years. The median age (interquartile) of 37. The median weight and height were 32 kg (IQR = 27–39) and 137 cm (IQR = 129–146). The proportion of children who were stunted and wasted were 4% and 3.0% respectively.

### Genotype and allele frequencies

The genotype and allele frequencies for CYP3A4*1B, *CYP3A5* (*3, *6, *7), *CYP2C19* (*2, *3, *17), *CYP2C9* (*2, *3) and *CYP2J2* (*7) among Rwandan children is presented in Table 1. There were no significant differences between the observed and expected genotypes frequencies according to the Hardy Weinberg Equilibrium. The most frequent variant was CYP3A4*1B (72.0%), followed by CYP3A5*6 (20.0%) and CYP3A5*3 (18.2%). CYP2C9*2 and CYP2C9*3 alleles occurred at the lowest frequency (0.2%).

**Table 1:** Genotype and allele frequencies for *CYP3A4*, *CYP3A5*, *CYP2C9*, *CYP2C19* and *CYP2J2* in the study population.

Variant allele	Allele frequency (%)	Genotype frequency N (%)	
CYP3A4 *1B	72.0	*1/*1	36 (7.8)
		*1/*1B	187 (40.5)
		*1B/*1B	239 (51.7)
CYP3A5 *3	18.2	*1/*1	311 (67.3)
		*1/*3	134 (29.0)
		*3/*3	17 (3.7)
CYP3A5 *6	20.0	*1/*1	294 (63.6)
		*1/*6	151 (32.7)
		*6/*6	17 (3.7)
CYP3A5 *7	10.8	*1/*1	369 (79.9)
		*1/*7	86 (18.6)
		*7/*7	7 (1.5)
CYP2C9 *2	0.2	*1/*1	460 (99.6)
		*1/*2	2 (0.4)
		*2/*2	0 (0)
CYP2C9 *3	0.2	*1/*1	460 (99.6)
		*1/*3	2 (0.4)
		*3/*3	0 (0)
CYP2C19 *2	12.4	*1/*1	355 (76.8)
		*1/*2	99 (21.4)
		*2/*2	8 (1.7)
CYP2C19 *3	0.1	*1/*1	461 (99.8)
		*1/*3	1 (0.2)
		*3/*3	0 (0)
CYP2C19 *17	20.2	*1/*1	291 (63.0)
		*1/*17	155 (33.6)
		*17/*17	16 (3.4)
CYP2J2*7	13.4	*1/*1	345 (74.7)
		*1/*7	110 (23.8)
		*7/*7	7 (1.5)

### Effect of genotypes on plasma praziquantel concentrations

The overall geometric means ± SD concentrations of PZQ, *trans*-4-OH-PZQ and *cis*-4-OH-PZQ in the study population were 318.4 ± 4.1, 8770.0 ± 2.2 and 571.5 ± 2.8 ng/mL, respectively. The overall geometric means ± SD concentrations of the PZQ MRs namely *trans*-4-OH-PZQ/PZQ and *cis*-4-OH-PZQ/PZQ were 27.5 ± 2.7 and 1.8 ± 2.9 ng/mL, respectively.

Comparison of PZQ, *trans*-4-OH-PZQ/PZQ and *cis*-4-OH-PZQ/PZQ concentration between the different genotypes are presented in Table 2. CYP2C9 *2, *3 carriers had significantly higher PZQ concentration and lower trans-4-OH-PZQ and cis-4-OH-PZQ MRs compared to children with CYP2C9*1/*1 genotype. CYP2C19 genotype was significantly associated with PZQ and *cis*-4-OH-PZQ/PZQ concentrations. Children with CYP2C19 *1/*17 or *17/*17 (ultra-rapid metabolizers) had the lowest PZQ concentration and children with *2, *3 carriers (intermediate and poor metabolizers) had the lowest PZQ concentration. Conversely the trans-4-OH-PZQ and cis-4-OH-PZQ MRs where highest among CYP2C19 *1/*17 or *17/*17, and those who were *2, *3 carriers had the lowest MRs. *CYP3A4* was also associated with cis MR, CYP3A4*1B carriers had a higher *cis*-4-OH-PZQ/PZQ MR compared with those who carried than CYP3A4*1/*1 genotypes. No significant effect of *CYP3A5* and CYP2J2*7 genotypes on PZQ and its MRs was observed.

**Table 2:** Comparison of the geometric means of PZQ concentration (ng/mL) and metabolic ratios *trans*-4-hydroxy-praziquantel/praziquantel and *cis*-4-hydroxy-praziquantel/praziquantel between CYP450 genotypes.

Genotype		N	PZQ GM ± SD	p-value	Trans-4-OH-PZQ/PZQ GM ± SD	p-value	Cis-4-OH-PZQ/PZQ GM ± SD	p-value
*CYP3A4*	*1/*1	36	292.9 ± 4.6	0.36	26.3 ± 2.4	0.39	1.3 ± 2.2	**0.04**
	*1B carriers	424	320.8 ± 4.1		27.6 ± 2.7		1.8 ± 3.0	
*CYP3A5*	*1*1	119	280.0 ± 1.1	0.13	27.5 ± 1.1	0.50	1.8 ± 1.1	0.40
	*3, *6, *7 carriers	341	333.1 ± 1.1		27.5 ± 1.1		1.8 ± 1.1	
*CYP2C9*	*1/*1	456	314.5 ± 1.1	**0.02**	27.8 ± 1.0	** < 0.001**	1.8 ± 1.1	**0.02**
	*2, *3 carriers	4	1356.3 ± 1.5		7.2 ± 1.3		0.6 ± 1.4	
*CYP2J2*	*1/*1	344	312.1 ± 1.1	0.30	27.1 ± 1.1	0.31	1.8 ± 1.1	0.30
	*7 carriers	116	388.3 ± 1.1		28.6 ± 1.1		1.7 ± 1.1	
*CYP2C19*	*1/*17, or *17/*17 (UM)	147	246.0 ± 4.1	** < 0.001**	38.5 ± 2.6	0.39	2.2 ± 2.8	**0.02**
	*1/*1 (EM)	205	309.5 ± 4.6		26.6 ± 2.7		1.8 ± 3.1	
	*2, *3 carriers (IM&PM)	108	477.6 ± 3.0		18.3 ± 2.4		1.3 ± 2.5	

*GM* geometric mean, *OH* hydroxyl, *PZQ* Praziquantel, *SD* standard deviation, *UM* ultra-rapid metabolizers, *EM* extensive metabolizers, *IM* intermediate metabolizers, *PM* poor metabolizers.

Significant values are in bold.

### Predictors of praziquantel plasma concentrations

Factors associated with plasma PZQ concentration is shown in Table 3. In the univariate linear regression analysis, age, weight, height, and genotypes *CYP2C9* and *CYP2C19* were significant predictors of PZQ concentration. In the multivariate model, after adjusting for age, height, weight and *CYP2C9*, *CYP2C19* genotypes remained significantly associated with plasma PZQ concentration. CYP2C19*2 and CYP2C19*3 carriers (intermediate and poor metabolizers) had a mean increase concentration of 0.185 ng/mL (95% CI 0.045–0.326) compared to those with CYP2C19*1/*1 (extensive metabolizers) (p value = 0.01). Ultrarapid metabolizers (CYP2C19 *1/*17 or *17/*17) had a lower mean PZQ concentration of 0.08 ng/mL (95% CI − 0.208 to 0.048) compared to extensive metabolizers but this was not statistically significant (p = 0.22). After adjusting for *CYP2C19*, age, height and weight, carriers of CYP2C9*2 or CYP2C9*3 had an increased mean PZQ concentration of 0.54 ng/mL (95% CI − 0.056 to 1.134, p = 0.08) compared to CYP2C9*1/*1 genotypes*.*

**Table 3:** Factors associated with plasma praziquantel concentration (ng/mL).

Variable		Crude Log mean difference (95% CI)	*p-*value	Adjusted Log mean difference (95% CI)	*p-*value
Sex	Female	Ref.			
	Male	0.032 (− 0.081 to 0.145)	0.58		
Age		0.042 (0.017 to 0.066)	**0.001**	0.007 (− 0.033 to 0.047)	0.73
Weight (kg)		0.012 (0.006 to 0.018)	** < 0.001**	0.007 (− 0.006 to 0.021)	0.30
Height (cm)		0.008 (0.004 to 0.012)	** < 0.001**	0.002 (− 0.008 to 0.012)	0.64
Wasting status (BAZ)	Not wasted	Ref.			
	Wasted	− 0.224 (− 0.552 to 0.105)	0.18		
Stunting status (HAZ)	Not Stunted	Ref.			
	Stunted	− 0.025 (− 0.141 to 0.092)	0.67		
*CYP3A4*	*1/*1	Ref.			
	*1B carriers	0.040 (− 0.171 to 0.250)	0.71		
*CYP3A5*	*1/*1	Ref.			
	*3, *6, *7 carriers	0.075 (− 0.053 to 0.204)	0.25		
*CYP2C9*	*1/*1	Ref.		Ref.	
	*2, *3 carriers	0.635 (0.028 to 1.241)	**0.04**	0.540 (− 0.056 to 1.134)	0.08
*CYP2J2*	*1/*1	Ref.			
	*7 carriers	0.035 (− 0.095 to 0.165)	0.60		
*CYP2C19*	*1/*1 (EM)	Ref.		Ref.	
	*2, *3 carriers (IM&PM)	0.188 (0.046 to 0.330)	**0.01**	0.185 (0.045 to 0.326)	**0.01**
	*17 (*1/*17 or *17/*17) (UM)	− 0.010 (− 0.229 to 0.030)	0.13	− 0.080 (− 0.208 to 0.048)	0.22

*BAZ* body mass index (BMI) for age Z score, *EM* extensive metabolizers, *HAZ* height for Age Z score, *PZQ* praziquantel, *IM* intermediate metabolizers, *UM* ultrarapid metabolizers.

Significant values are in bold.

### Predictors of praziquantel metabolic ratios

Predictors for the PZQ MR concentrations namely *trans*- and *cis*-4-OH-PZQ/PZQ are presented in Table 4. Age, height, weight, *CYP2C9* and *CYP2C19* were crude significant predictors of both trans and *cis*-4-OH-PZQ/PZQ MR. *CYP3A4* was also a crude predictor for *cis*-4-OH-PZQ/PZQ. In the adjusted model, statistically significant predictors for *trans*-4-OH-PZQ/PZQ MR included age, *CYP2C9* and *CYP2C19*. For every one-year increase in age, the mean concentration of *trans*-4-OH-PZQ/PZQ MR decrease by 0.034 (p value 0.01; 95% CI − 0.061 to − 0.007). CYP2C9 *2 and *3 carriers had significantly lower *trans*-4-OH-PZQ/PZQ mean MR compared to those with CYP2C9 *1/*1 genotypes. The mean trans MR was lower among CYP2C19 *2 and *3 carriers (p < 0.001) compared to non-carriers of the variant alleles. Children who carried CYP2C19 *1/*17 or *17/*17 had a significantly higher mean trans-4-OH-PZQ/PZQ MR of 0.136 (p-value < 0.001; 95% CI 0.049–0.223) compared to children who were *1/*1. *Cis*-4-OH-PZQ/PZQ concentration was significantly associated with *CYP3A4* and *CYP2C19* genotypes after adjusting for selected variables. CYP3A4*1B carriers had a mean increased mean cis MR of 0.165 (p value 0.04; 95% CI 0.010–0.320) compared to with CYP3A4 *1/*1. CYP2C19 *2, *3 carriers had a lower mean *cis*-4-OH-PZQ/PZQ MR compared to CYP2C19 *1/*1 (p value < 0.01). There was a borderline association between CYP2C9 genotype and *cis*-4-OH-PZQ/PZQ MR, in comparison with children who were CYP2C9 *1/*1, the mean MR was lower by 0.425 (p value = 0.06; 95% CI − 0.875 to 0.025) among children who carried CYP2C9 *2, *3 alleles.

**Table 4:** Factors associated with Praziquantel metabolic ratios (*trans-4-OH-PZQ/PZQ and cis-4-OH-PZQ/PZQ*).

Variable		Trans-4-OH-PZQ/PZQ MR	*p-*value	Trans-4-OH-PZQ/PZQ MR		Cis-4-OH-PZQ/PZQ MR	*p-*value	Cis-4-OH-PZQ/PZQ MR	*p-*value
		Crude Log mean diff. (95% CI)		Adjusted Log mean diff. (95% CI)	*p-*value	Crude Log mean diff. (95% CI)		Adjusted Log mean diff. (95% CI)	
Sex	Female	Ref.				Ref.			
	Male	− 0.048 (− 0.127 to 0.031)	0.24			− 0.045 (− 0.131 to 0.040)	0.30		
Age		− 0.036 (− 0.053 to − 0.019)	** < 0.0001**	− 0.034 (− 0.061 to − 0.007)	**0.01**	− 0.026 (− 0.044 to − 0.007)	** < 0.01**	− 0.020 (− 0.050 to 0.010)	0.20
Weight (kg)		− 0.007 (− 0.012 to − 0.003)	**0.001**	− 0.007 (− 0.017 to 0.002)	0.11	− 0.006 (− 0.011 to − 0.001)	**0.01**	− 0.006 (− 0.017 to 0.004)	0.22
Height (cm)		− 0.004 (− 0.007 to − 0.001)	**0.01**	0.006 (− 0.001 to 0.012)	0.10	− 0.003 (− 0.001 to 0.000)	0.06	0.004 (− 0.004 to 0.011)	0.35
Wasting status (BAZ)	Not wasted	Ref				Ref			
	Wasted	0.167 (− 0.062 to 0.397)	0.153			0.235 (− 0.013 to 0.483)	0.06		
Stunting status (HAZ)	Not Stunted	Ref				Ref			
	Stunted	− 0.066 (− 0.148 to 0.015)	0.11			− 0.023 (− 0.111 to 0.065)	0.60		
*CYP3A4*	*1/*1	Ref				Ref		Ref	
	*1B carriers	0.021 (− 0.126 to 0.168)	0.78			0.146 (− 0.013 to 0.305)	0.07	0.165 (0.010 to 0.320)	**0.04**
*CYP3A5*	*1*1	Ref				Ref.			
	*3, *6, *7 carriers	− 0.000 (− 0.090 to 0.090)	0.98			− 0.013 (− 0.111 to 0.084)	0.79		
*CYP2C9*	*1/*1	Ref		Ref.		Ref.		Ref	
	*2, *3 carriers	− 0.587 (− 1.010 to − 0.164)	** < 0.001**	− 0.451 (− 0.854 to − 0.047)	**0.03**	− 0.505 (− 0.964 to − 0.047)	**0.03**	− 0.425 (− 0.875 to 0.025)	0.06
*CYP2J2*	*1/*1	Ref				Ref.			
	*7 carriers	0.023 (− 0.068 to 0.114)	0.61						
*CYP2C19*	*1/*1 (EM)	Ref		Ref		Ref		Ref	
	*2, *3 carriers (IM & PM)	− 0.164 (− 0.261 to − 0.067)	**0.001**	− 0.161 (− 0.256 to 0.066)	**0.001**	− 0.168 (− 0.275 to − 0.060)	** < 0.01**	− 0.167 (− 0.273 to − 0.060)	** < 0.01**
	*1/*17 or *17/*17 (UM)	0.159 (0.070 to 0.247)	** < 0.001**	0.136 (0.049 to 0.223)	** < 0.01**	0.065 (− 0.032 to 0.163)	0.19	0.050 (− 0.047 to 0.147)	0.31

*BAZ* body mass index (BMI) for age Z score, *EM* extensive metabolizers, *HAZ* height for age Z score, *IM* Intermediate Metabolizers, *MR* metabolic ratio, *PZQ* Praziquantel, *UM* ultrarapid metabolizers.

Significant values are in bold.

### Association of Praziquantel concentrations and genotype with MDA-associated adverse events

**Figure 1:**
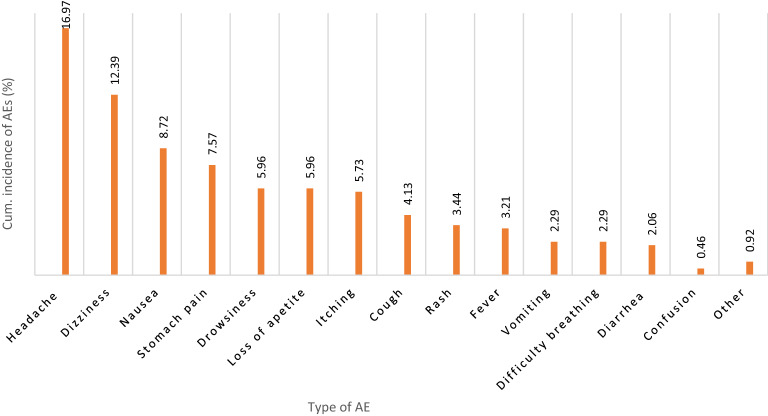
Type and cumulative incidence of MDA associated adverse events (AEs) during the 7-day follow-up.

A total of 436 children completed the safety 7-day post-MDA follow-up, of whom 36.0% (31.5–40.7%) reported at least one type of MDA-associated AE. The total number of AEs reported was 348, of which most occurred on the first day of MDA (83.0%) followed by day 2 (13.5%), and during days 3–7 of post MDA (3.5%). The severity of reported AEs was graded as mild, moderate, or severe, following the Common Terminology Criteria for Adverse Events (CTCAE) version 5.0^32^. The proportion of mild and moderate AEs were 92.2% (number of AEs = 321) and 7.5% (number of AEs = 26), respectively. Only one AE (0.3%) was severe. No potentially life-threatening or disabling AEs or death was observed. The cumulative incidences of specific type of adverse events are presented in Fig. 1. The most common AEs were a headache, dizziness, nausea, and abdominal pain.

Children who experienced MDA-associated adverse had higher geometric mean PZQ plasma concentration compared to children who did not, but the difference was not statistically significant (p = 0.15) as shown in Table 5. The geometric mean *cis*-4-OH-PZQ/PZQ MR was significantly lower among those who experienced adverse events compared to those who did not (p < 0.0001). There was also difference in crude and adjusted mean cis metabolic ratios among those children who experience adverse events compared to those who did not. After adjusting for age, weight and height, children who experienced adverse events had a mean decrease *cis*-4-OH-PZQ/PZQ concentration of 0.131 (p < 0.01; 95% CI − 0.223 to − 0.039) compared to children who did not experience adverse events. There was no statistically significant difference in *trans*-4-OH-PZQ/PZQ MR concentration between children who experienced adverse events compared to those who did not. Univariate followed by multivariate logistic regression analysis indicated no association between *CYP3A4*, *CYP3A5*, *CYP2C9*, *CYP2C19* and *CYP2J2* genotypes and experiencing MDA-associated adverse events.

**Table 5:** Comparison of the geometric means of PZQ, (ng/mL) and metabolic ratio (trans-4-OH-PZQ/PZQ and cis-4-OH-PZQ/PZQ) among those that experienced adverse events and those that did not.

Variables	Adverse event		P-value
	Yes	No	
PZQ GM ± SD (ng/mL)	367.9 ± 1.1	317.8 ± 1.1	0.15
Trans-4-OH-PZQ/PZQ GM ± SD	27.0 ± 1.1	26.8 ± 1.1	0.48
Cis-4-OH-PZQ/PZQ GM ± SD	1.4 ± 1.1	2.0 ± 1.1	** < 0.0001**

*PZQ* Praziquantel, *SD* standard deviation.

Significant values are in bold.

## Discussion

PZQ is metabolized by polymorphic enzymes and its plasma exposure and treatment outcome display wide inter-individual variability. Recent studies highlighted the need for more comprehensive studies of the PZQ metabolic pathway and PZQ pharmacogenetic studies in humans^20,23^. In this study, we investigated the effect of pharmacogenetic variations on PZQ pharmacokinetics and MDA-associated adverse events as well as the effect of pharmacokinetics on safety outcomes among school children who received praziquantel and albendazole preventive chemotherapy in Rwanda. Our major finding indicates that *CYP2C19* and *CYP2C9* genotypes as significant predictors of plasma PZQ concentration and its MRs (*trans*- and -*cis*-4-OH-PZQ/PZQ MR). Furthermore *cis*-4-OH-PZQ/PZQ MR concentration was significantly associated with CYP3A4*1B genotype. Another key finding was that *cis*-4-OH-PZQ/PZQ concentration was significantly associated with safety outcome (MDA-associated adverse event). There was no association between genotypes and adverse event following MDA.

PZQ is a racemic mixture *R*- and *S*-PZQ and is metabolized mainly by cytochrome P450 enzymes in the liver, specifically *CYP2C19*, *CYP2C9*, *CYP3A4*, *CYP1A2*, and *CYP2D6*^33,34^. We found significant associations between *CYP2C19* and *CYP2C9* genotypes with PZQ concentration and trans and *cis*-4-OH-PZQ MRs. Higher PZQ plasma concentration was observed in children with *CYP2C19* defective variant alleles compared to children carrying CYP2C19 *1/*1 and CYP2C19 *1/*17 or *17/*17 (ultra-rapid metabolizers). The mean of trans and cis-4-OH-PZQ/PZQ MRs were highest among ultra-rapid metabolizers (CYP2C19 *1/*17 or *17/*17) compared to those with carriers of *CYP2C19* defective variant alleles indicating that ultra-rapid metabolizers produce more metabolites compared to the other phenotypes. Although our study population received PZQ and albendazole as preventive chemotherapy without prior screening for the diseases, our finding is in line with a recent study among Schistosoma mansoni infected Tanzanian children who received PZQ therapy^20^. Our finding may indicate that *CYP2C19* is a major metabolic pathway for the formation of *trans*- and -*cis*-4-OH-PZQ metabolites. Li *et. al.*, found that *CYP1A2* and *CYP2C19* are the main enzymes responsible for metabolizing PZQ to its major metabolite, 4-OH-PZQ^35^. R-PZQ reportedly produces higher concentrations of *cis*- and *tran*s-4-OH-PZQ than its racemate, S-PZQ^36,37^. A recent study using recombinant and human liver microsomes reported that metabolism of R-PZQ was mainly catalyzed by *CYP1A2* and *CYP2C19*, whereas metabolism of S-PZQ was mainly by *CYP2C19* and *CYP3A4*^37^. Though polymorphic, functional defective variant alleles of *CYP1A2* are very rare in humans^38,39^. Hence the effect of CYP1A2 genetic variations in determining variability in plasma PZQ exposure could be minor.

Furthermore, we found significantly higher concentration of PZQ among children CYP2C9*2 or*3 carriers compared to those with CYP2C9 *1/*1. In the multivariable linear regression, after adjusting for potential confounders, the association between *CYP2C9* genotype was only significantly associated with *trans*-4-OH-PZQ/PZQ and *cis*-4-OH-PZQ/PZQ MRs and borderline associated with PZQ concentration. Our finding further supports that PZQ is mainly metabolized by CYP2C19 and to a lesser extent by *CYP2C9*^35,37^. Furthermore, a significant association between *CYP3A4* genotype and *cis*-4-OH-PZQ/PZQ but not PZQ concentration or trans MR was found. CYP3A4*1B carriers had significantly higher *cis*-4-OH-PZQ/PZQ MR compared to those with CYP3A4*1/*1. The effect of CYP3A4*1B on lumefantrine plasma exposure is reported previously^14^. Our finding indicates that compared to CYP2C19 and CYP2C9, the impact CYP3A4 and 3A5 genotype appears to be minor in determining variability in plasma PZQ exposure. The 4-hydroxylation of PZQ is reported to be the major metabolic pathway of PZQ, as evidenced by larger quantities of 4-OH-PZQ produced^40^.

In this study, we did not investigate the effect of *CYP1A1/2* and *CYP2D6* genotypes even though few studies reported their involvement in the metabolism of PZQ^33,34,37^. This can be considered as our study limitation, although functional CYP1A2 defective variant alleles are rare^38^. More studies are needed to investigate the influence of those genotypes on PZQ concentration especially when albendazole and PZQ are co-administered, it was reported that albendazole moderately inhibits the *CYP1A2* enzyme^41^. Furthermore, coadministration of PZQ and albendazole showed that the exposure of R-PZQ increased by 64.77% (AUC increased from 0.52 to 0.86 μg/mL h) but there was no significant difference in the AUC of S-PZQ^42^, this can because one or more enzymes are involved in PZQ and albendazole metabolism. This may improve the therapeutic outcome because of higher concentration of R-PZQ, the pharmacologically active enantiomer. On the other hand, this increase in R-PZQ concentration may affect safety even though, S-PZQ is the main contributor of PZQ associated side effects. Therefore, further studies are needed to evaluate treatment outcomes of the combination of PZQ and albendazole.

Non-genetic factors that influenced PZQ exposure in our study was age. We found age as a predictor of trans-4-OH MR, the main metabolite of PZQ in humans. This could be because of the rapid first pass effect (metabolism) of R-PZQ. Differences in the first pass effect based on age has been previously reported^43,44^.

Our finding indicates the relevance of plasma PZQ exposure and its MRs for MDA-associated adverse events. Though not significant, children who experienced adverse events had a higher mean plasma PZQ compared to those who did not. Interestingly there was a significant difference in mean cis MR among those the experienced adverse events compared to those who did not, this may indicate that those who experienced adverse events had a higher concentration of PZQ. Previous studies reported S-PZQ, the non-therapeutically active enantiomer, as the main contributor of the bitter taste of the drug and side effects such as nausea and vomiting^45–47^.

We found no significant association between the *CYP3A4*, *CYP3A5, CYP2C9*, *CYP2C19* and *CYP2J2* and MDA-associated adverse events safety treatment outcome. However, a previous study reported significantly higher incidence of adverse events among carriers of *CYP3A5* defective variant allele (67.0%) compared to those with CYP3A5 *1/*1 (33.0%)^20^. Any association of CYP3A genotype with adverse events could explained due to its relevance for S-PZQ metabolic pathway. CYP3A is the main enzyme that metabolizes S-PZQ^19,37^, which is responsible for the unpleasant taste of PZQ causing nausea and vomiting. As we quantified the racemate PZQ and not individual enantiomers, any association of genotype with enantiomers-specific adverse events could not be explored in our study.

Therefore, we recommend more studies investigating the association of CYP3A genotypes with S-PZQ plasma exposure and safety outcomes post PZQ exposure.

The study limitations include the following: We investigated the effects of CYP genotype on plasma concentration of praziquantel and metabolic ratio of the main metabolites 4-OH-PZQ, specifically trans-4-OH-PZQ. Since PZQ is administered as a racemic mixture of the two enantiomers, we measured total plasma PZQ concentration, but not R-PZQ and S-PZQ separately. Hence the effect of genotype on the enantiomer specific metabolism of PZQ were not assessed. Furthermore, we quantified the plasma concentrations of *trans* and *cis*-4-OH-PZQ, the main metabolites of R-PZQ, which is the therapeutically active form of PZQ^33,34^. Recent studies reported the importance of CYP3A for the metabolism of S‐PZQ to X‐OH‐PZQ^40,48^. As we did not measure X-OH-PZQ, the role of genotype on the metabolism of S-PZQ and its metabolic ratio were not explored.

## Conclusion

Our study highlights the importance of pharmacogenetic variation for PZQ pharmacokinetics and association of pharmacokinetics with safety outcomes. *CYP2C19* and *CYP2C9* were associated with plasma PZQ concentrations and *cis*- and *trans*-4-OH-PZQ MRs in Rwandan children. *CYP3A4* was only associated with the *cis*-4-OH-PZQ MR. Those who experienced adverse events had a significantly lower mean *cis*-4-OH-PZQ MR compared to those who did not. There was no association between *CYP3A4*, *CYP3A5, CYP2C9*, *CYP2C19* and *CYP2J2* genotypes and safety outcome. More studies are needed to evaluate the effect of pharmacogenetics on pharmacokinetics and pharmacodynamic especially among those who carry defective variant alleles of *CYP2C9* and *CYP2C19*.

## Data Availability

All data generated or analysed in this study are included in this published article, and the datasets are available from the corresponding author upon reasonable request owing to privacy and ethical restrictions from the authors.
